# Effective Biopesticides and Biostimulants to Reduce Aflatoxins in Maize Fields

**DOI:** 10.3389/fmicb.2019.02645

**Published:** 2019-11-21

**Authors:** Christina S. Lagogianni, Dimitrios I. Tsitsigiannis

**Affiliations:** Laboratory of Plant Pathology, Department of Crop Science, School of Plant Sciences, Agricultural University of Athens, Athens, Greece

**Keywords:** *Aspergillus* ear rot, aflatoxins, *Aspergillus flavus*, biological control, mycotoxins

## Abstract

The presence of ear rots in maize caused by *Aspergillus flavus* that are also associated with the production of aflatoxins has evolved into an increasing problem over the last few years. Since no commercial biological control products are still available to control *A. flavus* in maize in Europe, this study targets to the evaluation of six biopesticides/biostimulants (Botector®, Mycostop®, Serenade Max®, Trianum®, Vacciplant®, and zeolite) for the control of *A. flavus* and the derived aflatoxins in *in vitro* and maize field bioassays. Mycostop®, Serenade Max®, Vacciplant®, and zeolite reduced significantly *A. flavus* conidia production by 38.8–63.1%, and most of them were able to reduce aflatoxin B1 (AFB1) production in laboratory studies. Mycostop®, Trianum®, and Botector® were effective in reducing AFB1, *in vitro*. In the field, Mycostop® and Botector® treatments resulted in significant reduction of the disease severity (16.5 and 21.9%, respectively) and decreased significantly AFB1 content in maize kernels by 43.05 and 43.09%, respectively. For the first time, these results demonstrated the potential of commercial non-chemical products to suppress disease symptoms and aflatoxin content caused by *A. flavus* in maize under laboratory and field conditions.

## Introduction

Mycotoxins are toxic metabolites of low molecular weight that are produced by several species of mycotoxigenic fungi. A plethora of mycotoxins which are differing in their chemical structure have been identified, but all of them have the same common characteristics; they contaminate food and animal feed causing chronic toxicity and lead to more than 25% of agricultural products that are discarded annually (Bennett and Klich, 2003; CAST, 2003). One of the most common mycotoxigenic fungi is *Aspergillus flavus*, a predominant plant pathogen of maize (*Zea mays* L.) causing destructive plant diseases commonly known as ear rots and capable of contaminating maize kernels with aflatoxins (AFs). Toxigenic strains of *A. flavus* produce primarily the AFB1 and AFB2, although other mycotoxins (AFG1, AFG2, cyclopiazonic acid) can also be produced by the same species (Dorner and Cole, 2002; Dorner and Horn, 2007). AFs are worldwide one of the major threats to food quality and safety of the population feed. They are in first place (44%) as a reason for rejecting imports of various products in EU (RASFF/Rapid Alert System For Food and Feed for the European Union, 2008). Infection of maize by aflatoxigenic strains of *A. flavus* is favored by hot climatic conditions and the risk of aflatoxin biosynthesis is increased due to the dry and warm climate conditions combined with inappropriate storage conditions (Chulze, 2010).

Various strategies including chemical and biological control, development of tolerant varieties and control of insects that favor *Aspergillus* infection have been investigated in the effort to manage aflatoxins (AFs) in crops and agricultural products. Among them, biological control appears a very promising approach to control AFs at pre- and post-harvest level (Udomkun et al., 2017). In maize, the most susceptible stage for infection is during anthesis. Consequently, the most appropriate stage for application of biological or chemical plant protection products is this stage of ear development not only to protect wounds or plant surfaces, but also to give the biocontrol agents the ability to compete plant pathogens for space and nutrients (Vaughan et al., 2005; Dimakopoulou et al., 2008; Ponsone et al., 2011).

Numerous microorganisms including bacteria, yeasts, and non-toxigenic fungi of *A. flavus* have been evaluated for their ability to manage AF contamination in crops including maize, intending to reduce the impact of aflatoxigenic species (Yin et al., 2008; Ponsone et al., 2011; Mauro et al., 2018). Dorner (2004) and Atehnkeng et al. (2014) reported the efficacy of atoxigenic *A. flavus* strains in preventing AF contamination in maize field. Over time, several other effective non-toxigenic fungal strains have been commercialized like AF-X1® in Italy for aflatoxin management in maize (Mauro et al., 2018). In another study, it was reported the efficacy of two *Bacillus* strains in the control of *A. parasiticus* and aflatoxins production on pistachio (Siahmoshteh et al., 2017). Moreover, Chourasia and Sah (2017) pointed out the successful control of *A. flavus* and AF production with geocarposphere bacteria in peanuts in greenhouse experiments. In addition, Sivparsad and Laing (2016) showed that pre-harvest silk treatment with *Trichoderma harzianum* reduced disease severity and AF contamination caused by *A. flavus* in sweet corn, in greenhouse, and field experiments.

The use of biological agents and biostimulants for the control of *A. flavus* is a prerequisite for creating an Integrated Pest Management (IPM) in order to protect maize from AF contamination. Commercial biopesticides could offer an economically effective solution that will contribute to the exclusion of aflatoxigenic fungi from maize plants and the restriction of mycotoxin production with the help of an IPM system that will be friendly and sustainable for the environment. Mycotoxin control and reduction is crucial for food safety, animal welfare, human health reasons, and production economics (Bennett and Klich, 2003; CAST, 2003; Bosco and Mollea, 2012). In spite of the high contamination risk of maize by mycotoxigenic fungi and mycotoxins, biological control studies conducted on this particular crop are limited and most of them refer to *in vitro* results. This study suggests a biocontrol strategy based on commercial plant protection products to reduce AF contamination in maize fields. Therefore, the aim of the present study was: (1) to test the efficacy of six biopesticides/biostimulants, to inhibit conidiogenesis and aflatoxin production *in vitro*, and (2) to evaluate the potential of the most efficient products to reduce *A. flavus* infection and aflatoxin contamination of maize under field conditions.

## Materials and Methods

### Fungal Strains and Culture Conditions

Three *A. flavus* isolates were used in the experiments: A 6.10, D 1.3, and 12S. The isolates A 6.10 and D 1.3 originate from maize fields and pistachio orchards, respectively, in Greece and held in the culture collection of the Laboratory of Plant Pathology, Department of Crop Science, Agricultural University of Athens, whereas 12S originate from a cotton field in the USA. The isolates were mixed with glycerol (AppliChem, Darmstadt, Germany) to a final glycerol concentration of 25% (v/v) and stored at −20°C. The molecular characterization and the determination of aflatoxigenic efficacy of A 6.10, D 1.3, and 12S strains are described in our previous study (O’Donnell, 2000; Lagogianni and Tsitsigiannis, 2018).

### Biocontrol Products – Biopesticides/Biostimulants

Six products containing microorganisms or inorganic components with various modes of actions against a range of plant pathogens (Table 1) were used in bioassays: (1) zeolite, a microporous aluminosilicate mineral with special physicochemical properties, (2) Trianum®, a commercial product that contains the fungus *Trichoderma harzianum* and acts by inhibiting the infection and colonization of pathogenic fungi and inducing the plant defense system, (3) Botector®, a commercial product that contains yeasts of *Aureobasidium pullulans* with proven activity against *Botrytis cinerea* in grapes, (4) Mycostop®, a biological fungicide developed from the naturally occurring bacterium *Streptomyces griseoviridis* that provides biological protection against root infecting pathogenic fungi, (5) Serenade Max®, a commercial product that contains the bacterium *Bacillus subtilis* strain QST 713 with bio-fungicide/bio-bactericide action that stimulates natural plant defense mechanisms and demonstrates increased plant growth effects, and (6) Vacciplant®, which bases its action on activating the plant defenses thanks to the action of laminarine, a storage glucan from *Laminaria digitata*. All the above mentioned agents were initially tested *in vitro* and the most efficient were further evaluated in 2-year experiments under field conditions.

**Table 1:** Commercial biopesticides and biostimulants used in the present study, active ingredients and applied doses according to manufacturer’s instructions and company.

Product name	Active ingredient/biological agent	Applied dosage^a^	Company
Botector^®^	*Aureobasidium pullulans* strains	1 g L^−1^	Syngenta^®^
Trianum^®^	*Trichoderma harzianum*	3 g L^−1^	Koppert^®^
Mycostop^®^	*Streptomyces griseoviridis*	0.5 g L^−1^	Verdera^®^
Serenade Max^®^	*Bacillus subtilis* QST 713	4 g L^−1^	BASF^®^
Zeolite^®^	Mineral	10 g L^−1^	Olympos^®^
Vacciplant^®^	Laminarine	2 g L^−1^	Arysta^®^

a *Highest recommended dosage according to manufacturer’s instructions*.

### *In vitro* Evaluation of Biopesticides and Biostimulants on *Aspergillus flavus* Sporulation and Aflatoxin Production

The effect of the tested biopesticides and biostimulants on *A. flavus* sporulation and AFs production was initially studied *in vitro*. To conduct the bioassays, 40 g corn seeds (maize line N9, House of Agriculture Spirou, Athens, Greece) were surface-sterilized by immersing them in 10% NaClO for 10 min, washed briefly with sterile distilled water (SDW), placed in 70% ETOH for 3 min, and washed again with SDW for each biological product. The surface-sterilization of the seeds was carried out to avoid contamination from the seed surface saprophytes and keep the corn kernels alive. The seeds were not autoclaved to avoid the inactivation of the natural seed tolerance/resistance to *Aspergillus* infection provided by the plant immune system. Then, seeds for each treatment were placed into 250 ml capacity flasks containing each commercial product at the appropriate concentration according to the dose recommended by manufacturer’s instructions (Table 1). The flasks were shaken at 250 rpm for 30 min, then the solutions were discarded and corn seeds were kept at room temperature for 24 h. Then, seeds were artificially inoculated by adding in each flask 50 ml of *A. flavus* conidial suspension (10^6^ conidia ml^−1^) and shaking at 250 rpm for 30 min (Lagogianni and Tsitsigiannis, 2018). The suspension was removed and the flasks were placed at 28°C in the dark for 13 days to let the fungus produce AFB1. The presence of AFB1 in each sample was determined with thin layer chromatography (TLC) method, according to the following procedure: the seeds were grinded and 3 g of the fine powder were transferred into 50 ml falcon tubes, where 5 ml Tween 80 (0.01%) and 5 ml acetone were consecutively added. The samples were shaken at 150 rpm for 10 min and kept still for 5 min at room temperature; 5 ml chloroform were added and further shaken at 150 rpm for 10 min. The samples were passed through a filter paper and the flow-through collected into a new tube. The flow-through was centrifuged for 10 min at 3,000 rpm and the lower phase transferred into a new tube and kept overnight at room conditions to dry-out. Finally, 100 μl methanol were added and 10 μl of the sample spotted on a TLC plate (TLC Silica gel 60, Merck, Germany). TLC plate development and AFB1 detection were determined as mentioned above (Scott, 1995). The AFB1 that used as standard was purchased from Sigma-Aldrich.

To study the effect of the different tested biopesticides/biostimulants on the sporulation of *A. flavus*, each product was applied on corn seeds as described above and the seeds were placed in sterilized petri dishes (10 seeds per plate). Twenty-four hours later, one droplet of conidial suspension (10 μl of a 10^6^ conidia ml^−1^) of each *A. flavus* isolate (A 6.10, D1.3, and 12S) was deposited on each seed in the plate. Five days post inoculation, the 10 seeds of each plate were transferred in a new 50 ml falcon tube and 10 ml of sterilized distilled water was added. The samples were vortexed vigorously for 30 s and then the numbers of conidia were measured under a light microscope with the use of a Neubauer hemocytometer. The experiment was repeated three times, with 30 replicated maize seeds per treatment.

### Maize Field Experiments

Two-year experiments were carried out in the same experimental field of Agricultural University of Athens, Greece, in 2014 and 2015 crop seasons. Corn seeds (maize hybrid N9, House of Agriculture Spirou, Athens, Greece) were sown in the soil in April 2014 and 2015. Vacciplant® and zeolite were applied once whereas Mycostop®, Trianum®, and Botector® were applied twice by using a nozzle sprayer: the first application was carried out at the beginning of the flowering stage whereas the second one 7 days later. The applied dosages for each product are presented in Table 1, while no additional adjuvant or surfactants were used. The artificial inoculation was performed according to Lagogianni and Tsitsigiannis, 2018. Briefly, 5 ml of conidial suspension of *A. flavus* strain A6.10 (10^6^ conidia ml^−1^ in sterile ddH_2_0 containing 0.05 g L^−1^ Tween 80) were injecting in maize ears using a 10 ml capacity syringe with a needle. Three milliliter of the inoculum was injected through the silk into the top of each maize ear and 2 ml through the husk into the middle of the ear at each of four points. Both inoculated and mock inoculated ears were immediately covered with paper bags for 48 h to maintain high humidity and favor *Aspergillus* infection (Zummo and Scott, 1989). The experiments were performed with a factorial randomized block design with three blocks and six experimental units (Control+, Botector, Trianum®, Vacciplant®, Mycostop®, and zeolite) per block. Each experimental unit consisted of 30 replicated plants.

### Disease Assessment and AFB1 Analysis

Disease symptoms were assessed at the end of each growing season (60 days post inoculation), in September 2014 and 2015. Disease severity index was based on a visual scale from 1 to 7, considering the percentage of symptomatic kernels per ear (1 = healthy, 2 = 1–3%, 3 = 4–10%, 4 = 11–25%, 5 = 26–50%, 6 = 51–75%, and 7 = 76–100%) of infected kernels, respectively (Reid et al., 1999). Maize cobs were harvested and their kernels were detached and placed in a drying oven until their humidity reached 15–18%. Then kernels were homogenized using a grinder and 40 g of the fine powder were used for AFB1 analysis, following the Agra-Quant aflatoxin 4-40ppb ELISA kit protocol (Romer-Labs).

### Statistical Analysis

All experimental data were analyzed with SPSS statistical software (SPSS Inc., Chicago, IL, USA). Analysis of variance (ANOVA) was used to determine the effects of replication, treatment, year and their interaction on disease severity and AFs production in field experiments. In laboratory experiments, ANOVA was used to determine the effects of replication, treatment, and *A. flavus* isolate on conidia production. When a significant *F*-test was obtained for treatments (*p* ≤ 0.05), the data were subjected to means separation by Tukey’s honestly significant difference (HSD) test (Table 2).

**Table 2:** Analysis of variance for disease severity and aflatoxin (AFB1) quantity in maize plants artificially inoculated with *A. flavus* isolate A6.10, treated with commercial biopesticides based on *A. pullulans* (Botector^®^), *S. griseovirides* (Mycostop^®^), Zeolite^®^, *laminarine* (Vacciplant^®^), and *T. harzianum* (Trianum^®^) or not (positive control), under field conditions in 2014 and 2015.

Source	df^b^	*F* values^a^	
		Disease severity	AFB1
Replication	2	1.66	—
Treatment	5	23.30^***^	19.95^***^
Year	1	0.13	0.28
Replication × Treatment	10	3.02^*^	—
Replication × Year	2	0.28	—
Treatment × Year	5	1.40	0.29
Replication × Treatment × Year	10	0.26	—

a Symbols: ^*^ and ^***^ indicate significance at p ≤ 0.05 and 0.001 levels, respectively, according to the F test.

b *Degrees of freedom between groups*.

## Results

### Effect of Biological Products and Biostimulants on *Aspergillus flavus* Sporulation and Aflatoxin Production *in vitro*

**Figure 1:**
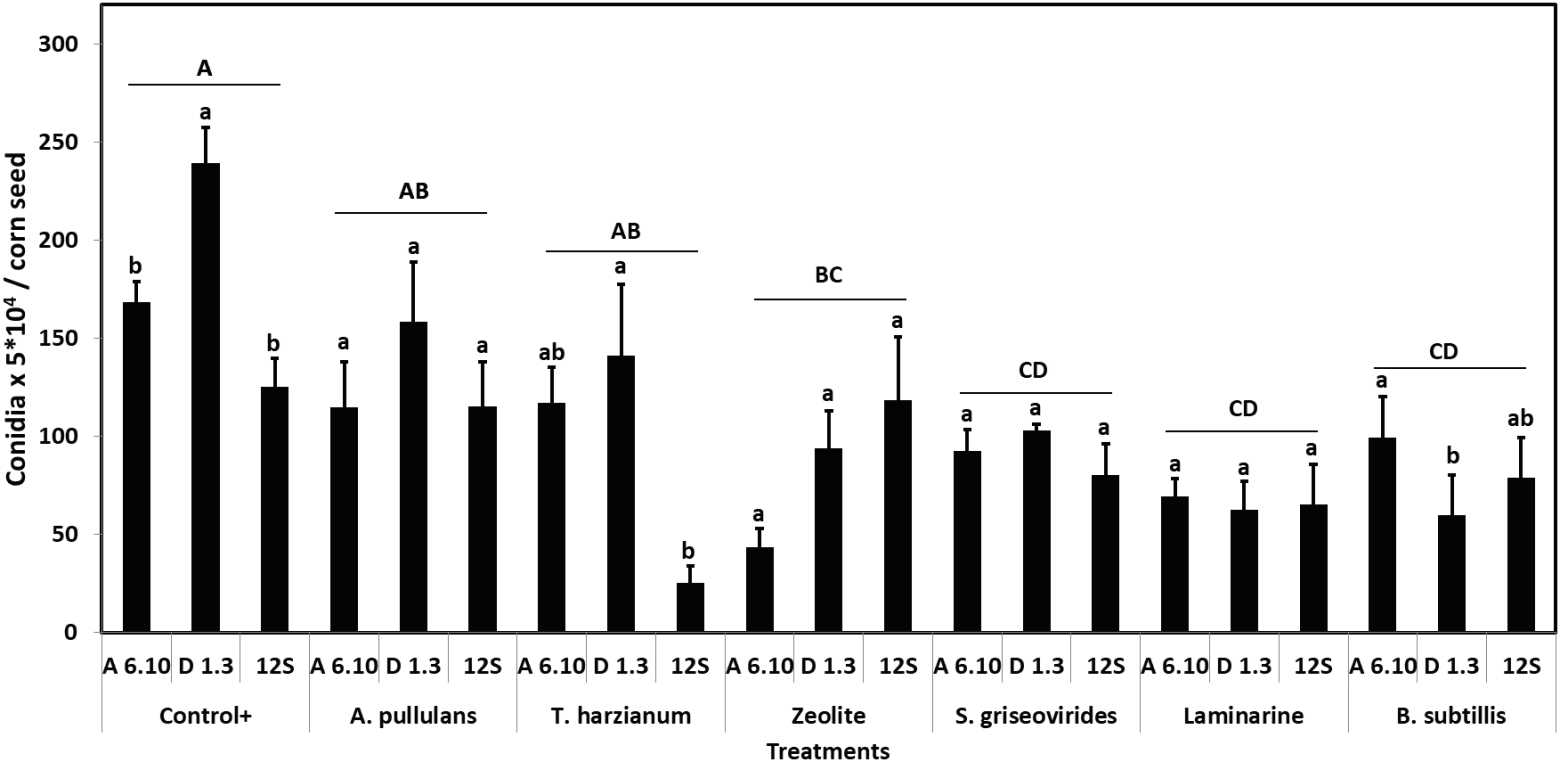
Mean numbers of conidia production of *A. flavus* by the strains A6.10, D1.3, and 12S in maize seeds treated with different commercial biopesticides/biostimulants. Within each treatment, columns with different lower-case letters differ significantly according to Tukey’s HSD test (*p* ≤ 0.05). Different upper-case letters indicate significant differences between treatments according to Tukey’s HSD test (*p* ≤ 0.05). Each column represents the mean of three measurements per isolate and vertical bars indicate standard errors of the means.

Among treatments, Vacciplant® was the most efficient in decreasing *A. flavus* sporulation *in vitro*, followed by Serenade Max®, Mycostop®, and zeolite leading to a reduction of conidia production by 63.1, 55.4, 48.2, and 52.1%, respectively. Botector® and Trianum® did not result in any significant reduction of fungal sporulation (Figure 1). Analysis of variance revealed that *A. flavus* isolates differed significantly in terms of sporulation (df = 2, *F* = 3.23, *p* < 0.05). Moreover, treatments effected significantly sporulation of the fungus *in vitro* (df = 6, *F* = 7.14, *p* < 0.001).

**Figure 2:**
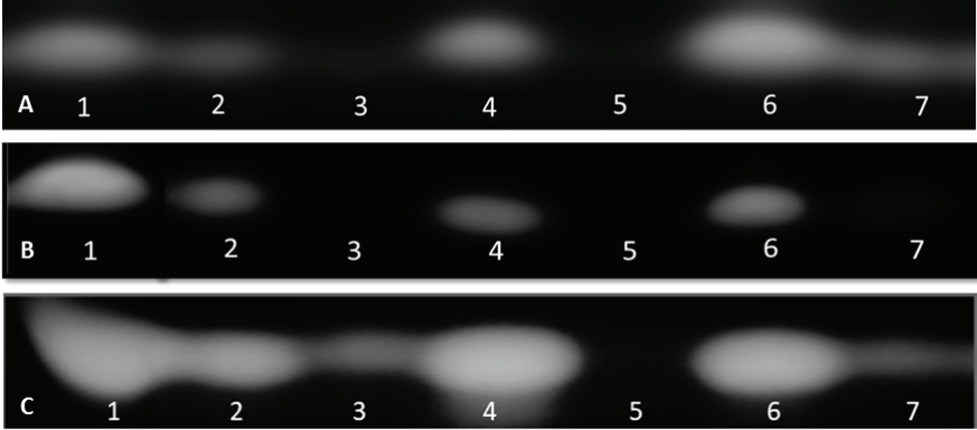
TLC detection of AFB1 in maize seeds treated with various biopesticides/biostimulants 13 days post their artificial inoculation with the toxigenic isolates D1.3 **(A)**, 12S **(B)**, and A6.10 **(C)** of *A. flavus* (1: non-treated seeds that served as positive control, 2: seeds treated with Vacciplant^®^, 3: seeds treated with Botector^®^, 4: seeds treated with Serenade Max^®^, 5: seeds treated with Mycostop^®^, 6: seeds treated with zeolite, and 7: seeds treated with Trianum^®^).

The capacity of the biological products and biostimulants to eliminate the aflatoxin production was also evaluated. AF was extracted from infected maize seeds and the extracts were developed by TLC. TLC tests showed that Mycostop®, Trianum®, and Botector® were very effective in reducing aflatoxin biosynthesis *in vitro*, produced by each toxigenic strain, whereas zeolite, Vacciplant®, and Serenade Max® did not provide a constant significant reduction in aflatoxin production (Figure 2).

Based on these results, Mycostop® contributed to the inhibition of the conidiogenesis and to a significant reduction in the AFB1 content for all the three tested *A. flavus* strains. Trianum® inhibited AF production but did not have any statistically significant effect to the conidia production. Vacciplant®, Serenade Max®, and zeolite did not lead to any reduction in the AFB1 content (Figure 2) but the inhibition of *A. flavus* conidia production was significant in the case of zeolite and Vacciplant®. Among the three strains, the conidiogenesis of D1.3 was not influenced significantly by the presence of the tested bioproducts except for the case of Serenade Max®.

### Suppression of Ear Rot Disease Symptoms and AFB1 Production in the Field by the Use of Biopesticides/Biostimulants

**Figure 3:**
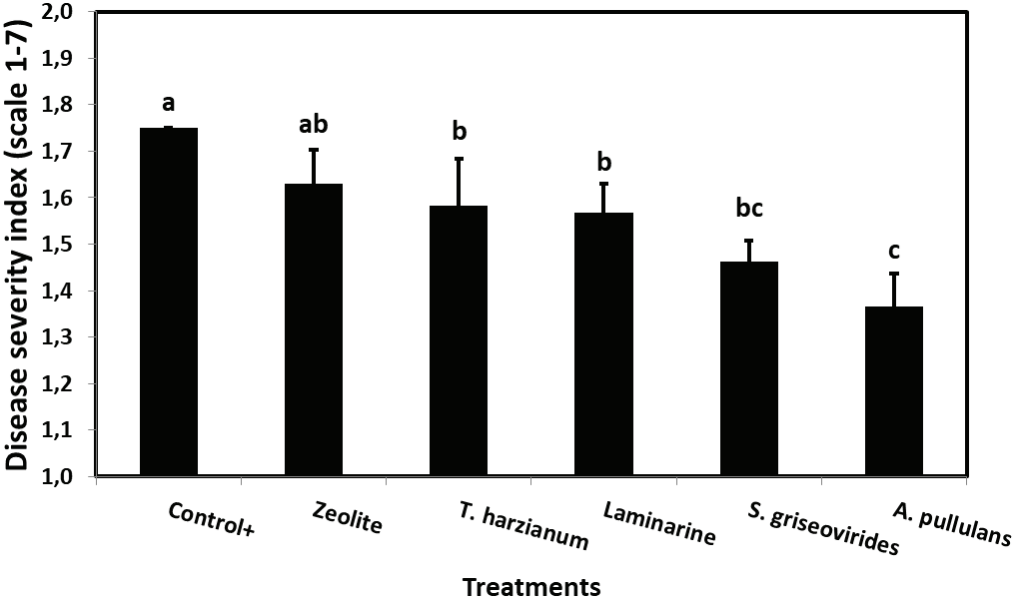
Mean *Aspergillus* ear rot severity indices on field grown maize plants treated with different commercial biopesticides/biostimulants and artificially infected by *A. flavus* maize strain A6.10. Columns followed by different letters are significantly different (*p* ≤ 0.05), according to Tukey’s HSD test. Vertical bars indicate standard errors of the means. The results represent the average *Aspergillus* ear rot severity for 2014 and 2015.

The toxigenic A6.10 maize strain, an isolate from Northern Greece, was used for the 2-year field experiments. Since Serenade Max® did not have a constant reduction of AFB1 in *in vitro* experiments, was not included in the field experiments. ANOVA revealed that neither experimental year nor the interaction between year and other experimental factors affected disease severity and AFB1 quantity significantly (Table 2). Therefore, data from 2-year experimentation (2014 and 2015) were combined and presented in Figure 3.

**Figure 4:**
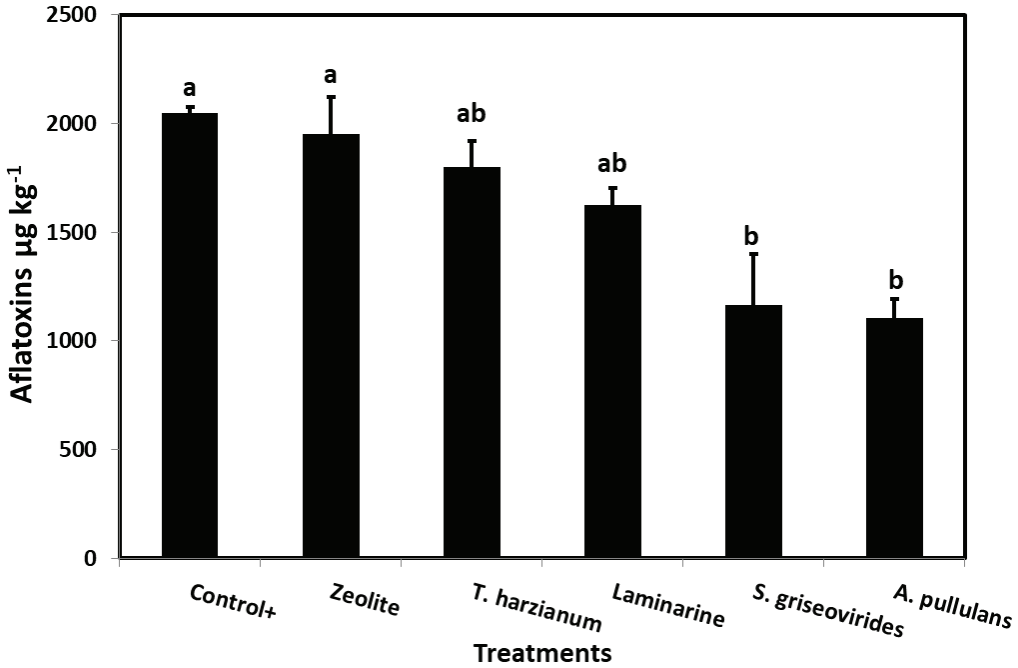
Mean AFs content (μg kg^−1^) in maize kernels from field grown plants treated with different commercial biopesticides/biostimulants and then artificially inoculated with *A. flavus* strain A6.10. Columns accompanied by different letters are significantly different (*p* ≤ 0.05) according to Tukey’s HSD test. Vertical bars indicate standard errors of the means. The results represent the average AFs content for 2014 and 2015.

In order to evaluate the disease severity of infected maize ears under field conditions after the application of the commercial biopesticides/biostimulants, a scale of 1–7 was used (Reid et al., 1999). The disease severity index in plants treated with Mycostop® and Botector® was significantly lower compared to the Control+ plants (by 16.5 and 21.9%, respectively), a fact that demonstrates the suppressive effect of the above mentioned products under field conditions (Figure 3). The observed decrease in symptom severity, in Mycostop® and Botector® treated plants was also associated with significantly lower AFB1 content in maize kernels, by 43.05 and 43.09%, respectively (Figure 4). Trianum® and Vacciplant® treated plants did not provide any statistically significant reduction on the AFB1 content, but offered a reduction in the disease severity whereas zeolite did not have any influence on either the disease severity or the AF content of maize ears.

## Discussion

Mycotoxins, especially aflatoxins, are one of the major worldwide threats to food quality and safety of the population feed. The public concern of pesticides and their residues as an emerging threat in food and environment have increased the interest in alternative methods for disease control both at pre- and post-harvest stages. In Europe, there is a lack of commercial products (biological or chemicals) to prevent AFs in maize despite the fact that EU sets very strict rules for the maximum limits of AFs in foods. Based on several studies and the impact of the climate conditions in the life cycle of mycotoxigenic fungi and mycotoxin production (Edlayne et al., 2009; Chulze, 2010; Russell et al., 2010; Battilani et al., 2012), a “biological” solution seems to be the only promising solution for the aflatoxin reduction combined with good agricultural practices, sustainable IPM strategies and agricultural precision technologies.

The use of certain bacteria, yeasts, and other antagonistic fungi to reduce AF contamination has been documented in maize, groundnut, and other crops (Nesci et al., 2005; Alaniz Zanon et al., 2013; Morteza et al., 2013; Zhao et al., 2014; Sivparsad and Laing, 2016; Siahmoshteh et al., 2017). In this study, six commercial biological products were evaluated for their ability to control *A. flavus* and aflatoxin production. Zeolite, Vacciplant®, Serenade Max®, and Mycostop® inhibited effectively the sporulation of all three *A. flavus* toxigenic strains *in vitro* by 48.2–63.1% with no statistically significant difference among the strains. In contrary, Botector® and Trianum® did not provide any significant inhibition in the sporulation of the three toxigenic strains when they were tested *in vitro*, but led to a significant reduction of AFB1 and *Aspergillus* ear rot severity in the field under a high *A. flavus* inoculum pressure per plant. Moreover, Mycostop® has the ability to inhibit the AFB1 content in maize field experiments, despite the fact that it did not suppress the ear rot severity by more than 10%. *In-vitro* tests do not resemble the natural environmental variation but they are always essential for the first screening of all plant protection products.

The two-year field experiment showed that when we applied the biological products Botector® (*A. pullulans*) and Mycostop® (*S. griseovirides*) twice during the silk stage, they were able to reduce AF production. The most effective commercial biopesticide was Botector® that showed an inhibition of *Aspergillus* ear rot severity by 22% and a significant reduction of aflatoxin content by 46%. *Bacillus* spp. and yeasts are growing at a faster rate than *A. flavus* and as a consequence, they can demonstrate a higher biocontrol efficacy during the first steps of incubation (Siahmoshteh et al., 2017). Based on several studies, the mode of action of *Bacillus* strains is the inhibition of mycelial growth and the antibiosis (Baysal et al., 2008; Zhao et al., 2014). Other studies, by Chan et al. (2003), mention that *Bacillus* strains have the same mode of action for other fungi except *A. flavus*, such as *Fusarium* sp., *Alternaria* sp., and *Phytophthora* sp. Mannaa et al. (2017) mentioned that some *Bacillus* strains reduced significantly the aflatoxin production in rice grains produced by *A. flavus* due to their volatiles. In our study, Serenade Max® (*Bacillus subtilis*) did not reduce the AFB1 content when tested by TLC.

Liu et al. (2013) reported that yeasts, such as *A. pullulans*, grow rapidly and as a result, deplete available nutrients and physically occupy the given space. After the colonization, other modes of action can play a significant role in concert with nutrient competition and niche exclusion to disclose decay management (Droby et al., 2000, 2009; Wisniewski et al., 2007). Moreover, the study of Ponsone et al. (2011) shows that some yeasts are able to deliver promising results against the grape rot by *Aspergillus* section *Nigri*. In accordance with our study, Dimakopoulou et al., 2008 mention that an *A. pullulans* isolation offered a significant reduction on *A. carbonarius* strain in grapes. Moreover, Prasongsuk et al. (2013) found that the components that lead to a reduction in AFB1 content are the aureobasidins.

Concerning Vacciplant® that is based on laminarine, Hu et al. (2011) mention that a specific concentration of laminarine could decrease the infection of peanut seeds by *A. flavus* as well as the contamination by AFB1. In the present study, we found that laminarine inhibits conidia germination but did not provide any protection against AFB1 biosynthesis.

In our studies, Trianum® (*T. harzianum*) led to a significant reduction of conidia production *in vitro*, but in the field experiments, did not significantly reduce the *Aspergillus* ear rot severity or the aflatoxin production. The mode of action of *T. harzianum* is based on its ability to successfully colonize a wide array of ecological niches (Schuster and Schmoll, 2010). The competitive exclusion of *T. harzianum* involves the utilization of limited resources, and as a result, the pathogen is unable to grow. Alamene (2015) found that *Trichoderma* strains from a commercial biocontrol product (Tusal)® can effectively inhibit toxigenic *A. flavus* species and AFB1 concentrations *in vitro* and *in planta*, to a level below that recommended by the European Commission of 15 ppb in peanuts. Gachomo and Kotchoni (2008) mention that two strains of *T. harzianum* and two strains of *T. viride* were found to efficiently suppress the growth of peanut molds and to significantly reduce aflatoxins (AFB1 and AFB2), contents in infected peanut kernels due to their extracellular enzymatic activities and mycoparasitism. Abdel-Megeed (2013) found that a *T. harzianum* strain provided significant suppression of AFB1 content by 91.2% in *in vitro* tests and Sivparsad and Laing (2016) found that *T. harzianum* colonizes the silk of sweet corn by inhibiting the *A. flavus* infection.

Finally, our results showed that zeolite has the capacity to inhibit conidia germination *in vitro*. These data are in agreement with the study of Savi et al. (2017) who present that the ion-exchanged zeolites with Li^+^ and Cu^2+^ have antifungal activity against *A. flavus*, including negative effects on conidia germination, hyphae morphological alterations, and inhibition of AFB1 production. Another study by Marković et al. (2015) indicates that zeolite can provide AFB1 adsorption. However, in our experiments, zeolite did not reduce AFB1 content neither in the field nor in *in vitro* tests. These results show that probably the application dose and application timing are crucial factors in the efficacy of zeolite *in planta*.

To date, there have been several studies demonstrating the efficacy of some microorganisms against *A. flavus* (Mannaa et al., 2017; Shakeel et al., 2018; Zeidan et al., 2018; Feng et al., 2019; Kagot et al., 2019; Mwakinyali et al., 2019; Peromingo et al., 2019). However, none of these studies have been conducted at field level and their tested microorganisms are not commercial formulations. Several factors can influence the efficacy of the biocontrol agents such as the cultivar response, the plant nutrition, the environmental variables, and the climate change. Furthermore, experiments about the right application and the appropriate number of application and dose could help to improve their efficacy against aflatoxins.

The European Commission suggests that, in southern Europe, climate change may lead to temperature increases of 4–5°C, in combination with increased drought periods (García-Cela et al., 2011; Battilani et al., 2012), conditions that will favor the production of aflatoxins in maize and other crops. An integrated approach of pre-harvest biological control, in conjunction with other post-harvest management strategies constitutes a very promising method for a long-term reduction in aflatoxin contamination in maize.

## Conclusions

The findings of these studies demonstrated for the first time, the potential of commercial non-chemical products (e.g., Botector® and Mycostop®) to suppress disease ear rot severity symptoms and decrease significantly AFB1 content in maize fields. Taking everything into account, the biological control of aflatoxigenic fungi, the control of insects, and the investigation on new maize aflatoxin tolerant hybrids/varieties along with effective chemical products (Lagogianni and Tsitsigiannis, 2018), disease forecasting models and decision support systems can lead to a successful IPM system in order to eliminate the aflatoxins problem in maize and other crops.

## Data Availability Statement

The datasets generated for this study are available on request to the corresponding author.

## Author Contributions

CL performed the experiments, analyzed the data, and wrote the manuscript. DT conceived the study, analyzed the data, and edited the manuscript.

### Conflict of Interest

The authors declare that the research was conducted in the absence of any commercial or financial relationships that could be construed as a potential conflict of interest.
