# Corrigendum: Effective Biopesticides and Biostimulants to Reduce Aflatoxins in Maize Fields

**DOI:** 10.3389/fmicb.2020.00029

**Published:** 2020-02-04

**Authors:** Christina S. Lagogianni, Dimitrios I. Tsitsigiannis

**Affiliations:** Laboratory of Plant Pathology, Department of Crop Science, School of Plant Sciences, Agricultural University of Athens, Athens, Greece

**Keywords:** *Aspergillus* ear rot, aflatoxins, *Aspergillus flavus*, biological control, mycotoxins

In the original article, there was a mistake in Table 1 as published. The company that provided the product named “Botector” with the biological agent *Aureobasidium pullulans* strains is not “Syngenta” company but “BIO-FERM.” Also, the company that provided the product named “Vacciplant” with the active ingredient *Laminarin* is not “Arysta” company but “GOEMAR.”

The corrected Table 1 appears below.

**Table 1:** Commercial biopesticides and biostimulants used in the present study, active ingredients and applied doses according to manufacturer's instructions and company.

**Product name**	**Active ingredient/biological agent**	**Applied dosage^**a**^**	**Company**
Botector^®^	*Aureobasidium pullulans* strains	1 g L^−1^	BIO-FERM^®^
Trianum^®^	*Trichoderma harzianum*	3 g L^−1^	Koppert^®^
Mycostop^®^	*Streptomyces griseoviridis*	0.5 g L^−1^	Verdera^®^
Serenade Max^®^	*Bacillus subtilis* QST 713	4 g L^−1^	BASF^®^
Zeolite^®^	Mineral	10 g L^−1^	Olympos^®^
Vacciplant^®^	Laminarin	2 g L^−1^	GOEMAR^®^

a *Highest recommended dosage according to manufacturer's instructions*.

In the original article, there was an error. The wrong company name was used in the **ACKNOWLEDGMENTS** section.

A correction has to be made to **ACKNOWLEDGMENTS** paragraph:

“The authors are grateful to BIO-FERM, Koppert, BASF, and GOEMAR for providing the commercial formulations used in this study.”

The authors apologize for these errors and state that this does not change the scientific conclusions of the article in any way. The original article has been updated.

